# Female Reproductive Health and Exposure to Phthalates and Bisphenol A: A Cross Sectional Study

**DOI:** 10.3390/toxics9110299

**Published:** 2021-11-11

**Authors:** Lidia Caporossi, Paola Viganò, Enrico Paci, Silvia Capanna, Alessandra Alteri, Giovanni Campo, Daniela Pigini, Mariangela De Rosa, Giovanna Tranfo, Bruno Papaleo

**Affiliations:** 1 INAIL—National Institute for Insurance against Accidents at Work, Department of Occupational and Environmental Medicine, Epidemiology and Hygiene, Via Fontana Candida 1, 00078 Monte Porzio Catone, Italy; e.paci@inail.it (E.P.); s.capanna@inail.it (S.C.); d.pigini@inail.it (D.P.); m.derosa@inail.it (M.D.R.); g.tranfo@inail.it (G.T.); b.papaleo@inail.it (B.P.); 2 Fondazione Scientific Institute for Research, Hospitalization and Healthcare Ca’ Granda Ospedale Maggiore Policlinico, Infertility Unit, Via M. Fanti, 20132 Milan, Italy; paola.vigano@policlinico.mi.it; 3 Scientific Institute for Research, Hospitalization and Healthcare San Raffaele Scientific Institute, Obstetrics and Gynecology Unit, Via Olgettina 60, 20132 Milan, Italy; alteri.alessandra@hsr.it (A.A.); campo.giovanni@hsr.it (G.C.)

**Keywords:** phthalates, reproductive health, women, bisphenol A, endocrine disrupters

## Abstract

The xenoestrogenicity of some plasticisers (phthalates and bisphenol A) is documented in the literature and may pose a risk to female reproductive health. The aim of this study was to assess exposure to six phthalates. This was achieved by measuring their respective metabolites (mono-ethylphthalate (MEP); mono-n-butylphthalate (MnBP); mono-n-ottylphthalate (MnOP); and monobenzylphthalate (MBzP)), as well as the sum of two of the diethyl-hexyl phthalate metabolites-(∑DEHP) and bisphenol A (BPA) in a female population with infertility problems, and by conducting a correlation analysis between infertility factors, work activities, and lifestyle habits, in order to formulate a causal hypothesis. A cross-sectional epidemiological study was carried out and women under 43 years of age were recruited from an assisted reproduction technology (ART) center; the sample of 186 women was given a specific questionnaire and a spot urine sample was collected. Phthalate metabolites and urinary BPA were analyzed by HPLC/MS/MS. The results showed significantly higher mean values for MEP in women with recurrent pregnancy loss (RPL) (820.5 ± 1929.5 µg/g of creatinine) and idiopathic infertility (230.0 ± 794.2 µg/g of creatinine) than in women with other infertility factors (76.9 ± 171.8 µg/g of creatinine). Similarly, for MnOP levels, women with idiopathic infertility (2.95 ± 3.44 µg/g of creatinine) showed significantly higher values than women with the other infertility factors taken together (1.35 ± 2.05 µg/g of creatinine). Women with tubal factors of infertility, RPL, and endocrine dysfunctions show higher values of DEHP (*p* = 0.032). Considering occupations, women working in commerce showed more than twice as much urinary BPA levels (1.10 ± 0.48 µg/g of creatinine) compared to women working in other industries (0.45 ± 0.35 µg/g of creatinine). The presence of significantly higher values of certain phthalates, DEHP in particular, especially in women with RPL and idiopathic infertility, suggests a possible involvement of these compounds as competing factors in reproductive issues. The study of sources of exposure suggested that the working activity in trade, as a casher in particular, represents a major one for BPA (*p* = 0.015).

## 1. Introduction

Infertility is a pathological condition with a multifactorial aetiology, and is not always identifiable. Exposure to external factors that may affect reproductive health, present in living and working environments, can be a significant source of risk, which may not always properly be assessed.

In particular, attention has been paid to certain compounds, classified as endocrine disruptors, with a molecular structure capable of interacting with hormone receptors, in particular with oestrogen and androgen receptors [1,2,3]. These chemicals belong to a number of classes of plasticizers, and are present in different production cycles and in daily-use products. This can represent a source of exposure and therefore a potential hazard.

Adverse effects on reproductive health associated with exposure to certain phthalic acid esters, i.e., a group of structurally similar molecules widely used since 1930, have been documented in the literature [4], specifically di-(2-ethylhexyl) phthalate (DEHP), diethyl phthalate (DEP), dibenzyl phthalate (DBzP), n-butyl phthalate (DnBP), butylbenzyl phthalate (BBzP), more recently din-octyl phthalate (DnOP), and diiso-nonyl phthalate (DiNP).

High-molecular-weight phthalates, such as DEHP, DnOP, and DiNP, are common constituents of polyvinyl chloride materials. They are used in flooring products, food packaging, and soft medical tools [5,6]. Low-molecular-weight phthalates, such as DEP, DnBP, and BBzP, are used as solvents and fixatives in cosmetics and personal care products [7]. The routes of exposure can be multiple: inhalation, dermal absorption, as well as ingestion in case of food contamination. Urine excretion is the main elimination route as phthalates can be rapidly metabolized to monoesters and oxidation metabolites [8]. Therefore, urinary determination of phthalate metabolites has been widely used as an exposure biomarker in human studies. The excretion peak of concentration is around 4 h after exposure, with a complete excretion within 24 h [9].

The data available in the literature suggest a distribution of these endocrine disruptors of interest in both living and specific working environments, resulting in a detectable dose in human biological fluids. Exposure of the general population to these chemicals has been demonstrated by biomonitoring studies in both the United States and Europe [10,11,12].

Women in reproductive age seem to be more exposed to phthalates than men. This is probably due to the use of cosmetic and personal care products daily [7,13]. In females mice, a chronic exposure to high levels of some phthalates (DEHP and DnBP, in particular) may cause embryotoxicity or fetotoxicity [14,15]. Nevertheless, studies in humans focused on the cumulative exposure to phthalates produced conflicting results on the relationship between phthalate exposure and pregnancy loss [16,17,18].

Furthermore, some studies have shown that this type of exposure could be linked with a decreased antral follicle count [19], as well as lower numbers of retrieved oocytes, mature oocytes, fertilized oocytes, and high-quality embryos in assisted reproduction technology (ART) cycles [20,21].

Similarly, numerous studies in the literature have documented the endocrine disrupting action of bisphenol A (BPA), a compound with a chemical structure similar to 17-β-estradiol which can bind estrogen (α, β) receptors [22,23,24]. BPA is a monomer at the basis of polycarbonate plastics and a component of epoxy resins; it is used in numerous applications. Humans are exposed to BPA mainly by ingestion and dermal absorption [25]. Exposure may occur in occupational activities [26] or, primarily, because of migration into food from plastic containers [27,28]. The main part of the absorbed dose is eliminated from the body within 24 h by urine excretion [29].

While experimental studies, both in vivo and in vitro, agree in underlying the adverse effects of BPA on the reproductive process, with increased oocyte aneuploidy [30], chromosome segregation [31], and effects on meiotic segregation [32], results in human studies are conflicting.

Some authors found a negative association between BPA levels and estradiol concentrations [33,34] or the number of oocytes retrieved in ART cycles [35]. However, a study carried out in a wide population found no associations between BPA concentration and ovarian stimulations or pregnancy outcomes in women undergoing in vitro fertilization [36].

In order to understand what source of exposure could represent a health risk, studies were carried out in order to monitor the occupational settings [37,38]. Among phthalates, the majority of the studies investigated the possible DEHP exposure [37], even if its use is presently restricted by law. The plastic industry is by far the most involved in this type of exposure, both for phthalates [39] and for BPA [40].

The urinary levels detected in exposed workers to phthalates, in recent studies, are showed in Table 1.

Overall, the investigations conducted so far showed significantly higher urinary levels in exposed workers, compared to controls, confirming the need to evaluate carefully the work environment as a possible source of risk.

The occupational BPA exposure was investigated in cashiers [52] where the median concentration of BPA was 8.92 µg/L, i.e., more than twice higher compared to a control group (3.54 µg/L). In industries of thermal papers, paints, composites, and tractors [53], the urinary BPA levels in post-shift samples were up to 100–170 µg/L higher than pre-shift concentrations; peaks of 1000–1500 µg/L of BPA were recorded in workers of the coating machine in a thermal paper factory. In plastic industries, a stable presence of urinary BPA (average of 3.24 µg/L) was found in exposed workers [40].

The exposure to composite resins containing BPA in dental materials was evaluated but it seems to be insignificant [54].

Other investigated workers were represented by construction painters [55], and high levels of all biomarkers of BPA were found in the urine of mid-coat applicators.

A clinically infertile population of women may be more vulnerable to the toxicity of these chemicals than the general population, as they are already compromised from a reproductive standpoint [56]. Therefore, the identification of situations, both in workplaces and in a daily environment associated with a possible phthalate exposure, represents an important step to understand the main route of exposure and to promote the reproductive health.

The aim of this study was to assess whether exposure to phthalates and BPA can be correlated with specific reproductive disorders in a female population with manifest reproductive difficulties, and to investigate possible sources of exposure.

There is interest to confirm and extend the significance of the published results in the literature and to clarify controversial areas of research regarding the link between female reproductive health and exposure to phthalates and BPA.

## 2. Materials and Methods

### 2.1. Population Sample

The study protocol was evaluated and approved by the Institutional Review Board of the IRCCS San Raffaele Scientific Institute in Milan (identification code 73/INT/2017).

A total of 274 women considered underwent an ART treatment from February 2018 and March 2019 at San Raffaele Hospital (Milan, Italy). The sample size was reduced to 186 eligible subjects after considering exclusion criteria showed in Table 2. The parameters used in the statistical processing were also described as confounders and, therefore, were considered for the adjustment of the data.

Furthermore, an ad hoc questionnaire was set up for the collection of anamnestic information and life and working habits. All subjects completed the clinical anamnesis questionnaire under the guidance of well-trained investigators. The collected data included: life habits (such as smoking or alcohol drinking), diet, use of plastic containers to store fat foods, occupational activity, and clinical history (with specific focus on endocrine problems).

A spot urine sample was collected for the dosage of metabolites of the plasticizers of interest

### 2.2. Analysis of Biological Samples

Urinary analysis of phthalates involved the use of the previously validated and published HPLC/MS/MS method [57], which involves an initial sample pre-treatment step and a subsequent instrumental analysis step. BPA was analyzed using the method described in a further published paper [58], again using an API 4000 HPLC/MS/MS.

Samples were analyzed in duplicate. The concentration of each analyte was determined by calculating it from the linear regression equation identified with the calibration curve for the individual analyte, expressed in µg/L urine. This value was then normalized to the micrograms of creatinine determined in the same urine sample.

Determination of creatinine values was conducted by applying Jaffè’s method, using the alkaline picrate test with detection by UV/Visible spectrophotometry at a 490-nm wavelength [59]. Samples with creatinine concentrations greater than 3.0 g/L or less than 0.3 g/L were discarded, and the corresponding subjects were excluded from the study sample, as specified by the World Health Organization [60] and recommended by the American Conference of Governmental Industrial Hygienists (ACGIH) [61]. Indeed, values outside the normal range may represent specific disease situations or excessive dilutions, which would lead to potentially erroneous evaluations.

### 2.3. Statistical Analysis

Statistical analysis was performed with the software SPSS^®^ 25.0 (IBM, Armonk, NY, USA). Descriptive analysis was conducted for the population characteristics, along with the distributions of urinary metabolites levels. Values of phthalate metabolites below the limit of detection (LOD) were set to LOD/2. Data were not normally distributed; therefore, the results were logarithmic transformed to better adapt the data to the study design and to obtain a normal distribution. The urinary concentrations of phthalates were analyzed with parametric methods to identify differences between groups in terms of working activities, demographics, and habits. The ANOVA test, with a significance of 0.05, was used to understand the main potential sources of chemicals in living environment (the use of plastic containers for food storage; eating habits, such as taking products containing phytoestrogens, from soy to canned food; the use of enamels; and the use of hair sprays and perfumes) and to identify the most exposed working area. The same test also helped to highlight the differences in urinary metabolite levels linked to infertility factors. Confounding factors used in the statistical elaboration are reported in Table 2.

## 3. Results

Table 3 presents the characteristics of the sample of recruited women while the infertility factors identified in the sample are presented in Table 4.

Table 5 presents the results of the urinary assays of the endocrine disruptors of interest in the study.

Assay data for mono(2-ethylhexyl) phthalate (MEHP) and mono(2-ethyl-5-hydroxyhexyl) phthalate (MEHHP), as well as metabolites of the same phthalate (DEHP), are presented as the molar sum of the two.

A stratification of the data according to infertility factors is presented in Table 6.

Our findings show statistically significant differences among the groups, particularly for DEHP (*p* = 0.032). Furthermore, women with tubal factors of infertility, RPL, and endocrine dysfunctions show higher values. By comparing some specific infertility factors to all others, other significances emerged.

Focusing on the cases of RPL and idiopathic infertility, the mean urinary concentration values of MEP were 820.45 ± 1929.53 µg/g of creatinine (RPL) and 230.53 ± 794.17 µg/g of creatinine (idiopathic infertility). These values, compared to the average value of all other infertility factors taken together of 76.93 ± 171.77 µg/g of creatinine, show statistically significant differences (F Test *p* = 0.047). Subsequently, a Tukey comparison test was performed which confirmed significant differences between RPL and all other factors (*p* = 0.035). This is even more alarming given the high levels of exposure that can be detected, due in large part to the presence of DEP in many cosmetic formulations and everyday products.

A similar situation applies to MnOP, where idiopathic infertility has significantly higher values (2.95 ± 3.44 µg/g of creatinine) than the other infertility factors taken together (1.35 ± 2.05 µg/g of creatinine) (*t* test, *p* = 0.021).

Finally, MnBP presented higher values, which are also statistically significant (*t* test *p* = 0.044), in the case of women with ovulatory and endocrine dysfunctions (48.80 ± 71.76 µg/g of creatinine) than in the case of the other infertility factors (21.91 ± 15.72 µg/g of creatinine).

A subsequent stratification was carried out to highlight possible occupations potentially involved in exposure to phthalates and BPA (Table 7). Transforming the data into natural logarithm and applying the ANOVA test showed a significant difference in the groups for BPA. In particular, the group of women working in commerce shows significantly higher urinary BPA values (mean value 1.10 ± 0.48 µg/g of creatinine) than women working in all other production sectors (mean value 0.45± 0.35 µg/g of creatinine).

For MnOP levels, similarly, there is a significant value in women in commerce (3.11 ± 1.91 µg/g of creatinine) and in women working in “other sectors” (5.09 ± 3.72 µg/g of creatinine) compared to women in all other occupations (1.73 ± 1.54 µg/g of creatinine).

Finally, a stratification for possible sources of exposure in living environments was carried out. A significant difference among the various classes identified with respect to the habit of storing fatty food in plastic containers and the urinary dosage of MnBP (test ANOVA *p* = 0.028). This difference does not translate into a direct relationship with respect to frequency of use, but produces a non-linear trend (daily: 17.90 µg/g of creatinine, weekly: 31.04 µg/g of creatinine, monthly: 29.64 µg/g of creatinine, never 20.65 µg/g of creatinine), so the figure is not significant in identifying the source of exposure.

## 4. Discussion

The way that phthalates may influence pregnancy outcome has not been fully clarified. Some published studies suggested the association of phthalates with increased risk of spontaneous pregnancy loss, although these results were inconclusive and often conflicting.

A cohort study [16] involving 3220 pregnant women in China showed an increased risk of embryo loss (6–10 gestational weeks) for high concentrations of MEP (OR = 1.99) and of DEHP metabolites (OR = 2.19). Conversely, an association with fetal loss at 11–27 gestational weeks was found only for high MEHHP concentrations (OR = 2.41).

Other researchers [19] observed similar data. Specifically, the molar sum of DEHP metabolites was positively correlated with pregnancy loss in women undergoing ART. In a case–control survey on 260 patients with RPL and 203 controls [62], the risk of RPL was found to be strongly related to the highest quartiles of DEHP (*p* < 0.05), corresponding to urinary concentration levels (as geometric mean) higher than 0.27 µg/L. Unfortunately, this study did not provide the creatinine-corrected results; however, a comparison with our findings (DEHP 20.92 µg/g of creatinine) highlights that the population studied herein has a markedly higher exposure, which further justifies the significance that emerged.

Among women undergoing ART, a diminished pregnancy success was found when urinary concentration of specific chemicals was higher. In particular, women in the highest quartile of DEHP level had, on average, lower probabilities of implantation (−22%), clinical pregnancy (−24%) and live birth (−38%) compared to women of the lowest quartile [63]. High exposure to MnBP and MEHP was statistically significantly associated with a lower likelihood of pregnancy, and the same result was obtained with cumulative exposure of different phthalates (MEP, MnBP, and mono-n-pentylphthalate; MEHP, MEHHP, MiNP, MBzP, and mono(2-ethyl-5-carboxypentyl)phthalate) [64].

Unexpectedly, Toft et al. [65] found conflicting results, with a positive association between MEHP concentration and subclinical embryo loss but an inverse correlation between MEHP level and clinical pregnancy loss. These data would suggest a possible greater susceptibility to the toxicity of these substances in an early gestational age.

The North Carolina Early Pregnancy Study [18] presented opposite results: the summation of DEHP metabolites was significantly correlated with reduced odds of pregnancy loss (<6 gestational weeks) and none of the phthalates was related to clinical pregnancy loss (6–25 gestational weeks).

Liao et al. [17] carried out a case–control survey in China with 103 patients with RPL and 76 controls. After a dosage of 11 urinary metabolites, a borderline significance for DEHP and MEP levels was observed while DnBP metabolites were associated with an elevated risk for RPL (OR = 2.85). While looking for a comparison of urinary levels between these findings and the results presented herein, we noticed the same order of magnitude for MnBP levels, but no significance in the association with RPL. While, for DEHP and MEP, our results showed significantly higher values, by two orders of magnitude. These findings suggest the need of geographically homogeneous comparisons as the sources of exposure and lifestyle habits can be very different in different areas.

Investigations aimed to evaluate the possible effects of exposure due to lower-molecular-weight phthalates showing an increased risk of pregnancy loss for higher (from first to fourth quartile) urinary concentrations of MEP, MnBP, and MiBP, in a cohort of 132 women [66]. The comparison between the urinary levels of this survey and our findings showed the same order of magnitude for MEP (median of 15.6 µg/g of creatinine vs. 13.26 µg/g of creatinine) and a three-times-higher concentration for MnBP (median 45.4 µg/g of creatinine vs. 16.29 µg/g of creatinine). Similar findings were obtained by He et al. [67] in a case–control study of 123 women with pregnancy loss and 148 controls. Prenatal phthalates exposure contributed to an increased risk of abortion, in particular for the mono-methyl-phthalate (MMP) (OR = 2.85, 95% CI 1.34–6.05, in the fourth quartile of concentration).

Discrepancies among findings might be due to different characteristics of the studies, including sample size, study design, and inclusion criteria for recruited women. Based on a meta-analysis considering 4713 participants (including 651 cases and 4062 controls), the only phthalates with significant correlation with spontaneous pregnancy loss were MnBP (OR = 1.34) and DEHP (OR = 1.79). However, the same authors called for caution in the interpretation of their results owing to the limitation of published selected articles (only 8) [68].

Reference values have been proposed for phthalates and BPA for the general population. In consideration of the geographical variability that was found, those proposed for an Italian population were taken into consideration [69]. The following average results were reported: MEP 72.9 ± 355.91 µg/g of creatinine; MnBP 20.26 ± 56.21 µg/g of creatinine, MBzP 14.74 ± 53.19 µg/g of creatinine; MEHP 3.37 ± 5.70 of µg/g of creatinine; and MEHHP 12.74 ± 15.29 µg/g of creatinine. Other researchers [70] identified a mean value of 4.18 ± 1.84 µg/g of creatinine for BPA. A comparison of our results with these reference values highlighted an average concentration of MEP which was twice as high, while the other phthalates concentrations were of the same order of magnitude. For BPA, the urinary levels revealed in this study were lower than the reference value.

The results of our study confirm previous data in the role of DEHP as a toxicant for reproduction. Higher values were found in women with RPL, tubal factors, and endocrine dysfunctions. From the comparison with the other published studies, women under investigation showed a higher average level of DEHP, sometimes of two orders of magnitude; this finding underlines how, even with a law restriction, the DEHP exposure is still to be monitored because the concentrations are still relevant and the health effects could be considerable.

Regarding the lower molecular weight phthalates, our results suggest a possible role of MEP in the RPL cases, endorsing what has been previously published [66]. Furthermore, significantly higher levels of both MEP and MnOP emerged for idiopathic infertility cases and of MnBP in subjects with ovulatory dysfunctions.

Of particular interest, the mean urinary MEP levels were 2 to 10 times higher in the group of idiopathic infertility and RPL women, respectively, compared with the mean value in women with all the other infertility factors. MnOP levels also showed an approximately double value for women with idiopathic infertility compared with all the other subjects. As these substances have a xenoestrogenic action, the data suggest that this type of exposure may be directly involved in their reproductive problems.

To the best of our knowledge, no surveys have been conducted to evaluate a correlation between exposure to phthalates and idiopathic infertility in the female population. Few articles evaluated this issue in a male population, in order to understand the possible role of chemicals in the semen quality [71,72,73]. Our findings related, in particular, to MEP and MnOP exposure provide important information as a promising step for further research in more targeted epidemiological studies on female subjects.

In the literature, a significant link between embryo implantation rate and oocyte count [74,75] and urinary BPA concentration was found. BPA exposure was associated with diminished ovarian reserve and gynecological health risks [76]. These results suggest a role of BPA similar to other plasticizer, at an average concentration level between 1.00 and 2.70 µg/g of creatinine. These particular effects did not emerge in the present study where the overall mean value of urinary BPA was 0.71 µg/g of creatinine. Focusing on women with reduced ovarian reserve, a previously published paper [74] proposed the role of BPA as toxicant, with an average level of 1.89 µg/g of creatinine, while our results showed a BPA concentration which was significantly lower (average value = 0.69 µg/g of creatinine) without statistical significance compared to other women groups.

The low urinary concentration levels of our study sample (only 57% of urinary samples were >LOD) could represent a possible explanation for this findings. Recruited women seems to be significantly less exposed to BPA than those analyzed in literature, which could lead to the possibility of less noticeable health effects.

Regarding the sources of exposure [77], application of body washes, creams, skin care products, and cosmetics was shown to increase urinary concentrations of specific phthalates, such as DEHP, MEP, and MnBP. A secondary route of exposure could be identified in the assumption of certain medications [78], particularly for MnBP and DEHP exposure. Nevertheless, our findings did not detect exposure routes of major concern in living environment and daily habits.

Data stratification revealed a significant association between the habit of using plastic containers for storing fatty food and MnBP levels. However, these findings did not correspond to a direct relationship with the frequency of use, but produced a non-linear trend, which is less suggestive for the identification of a possible source of exposure. The main routes of BPA exposure are oral (for food contamination) [79] and dermal, especially in the case of occupational exposure to thermal paper [53,80]. The use of skin care products can sometimes increase the dermal absorption up to 100 fold, due to mixtures of dermal penetration-enhancing chemicals [79].

Stratifying the results for the different occupations clearly showed a significant relationship between working in the commercial sector and BPA levels. This can be explained by the daily contact with thermal paper of receipts as source of BPA, as documented in the literature [53,80].

This result confirms the need to investigate the sources of exposure also in epidemiological investigations. Particularly, understanding the possible exposures in the workplace is important, because the risk of identifying significant exposures, occurring over a long period, requires specific interventions to protect the health of the workers.

## 5. Conclusions

This study investigated the possible relationship between infertility factors and urinary dosages of certain phthalates and BPA, with a specific focus on the possible identification of sources of exposure.

The results of the investigation supported the theory that phthalate exposure might increase the risk of pregnancy loss, especially for DEHP and DEP.

An emerging finding is represented by the correlation between idiopathic infertility and the levels of MEP and MnOP. Furthermore, no previous research had highlighted this possible association. If confirmed, these data would require attention, particularly for MEP, whose urinary dosages are particularly high.

Currently, DEP, the parent phthalate of MEP, is not included among the compounds under restriction or authorization regulations in Europe, because data on humans are still considered limited. Nevertheless, emerging evidence emphasizes the requirement of a greater attention to identify its possible involvement in reproductive health diseases.

Our results regarding the occupational activities confirm what was already known in the scientific literature. Dermal contact with the thermal receipt paper represents an occupational significant exposure to BPA in our female population too. A specific restriction on the use of BPA in thermal receipt paper has been introduced on a European level, starting from January 2020, to protect workers.

The use of a single-spot urine sample could represent a limitation of the present study. The urinary concentration levels, on average, reflect the exposure occurred in the previous 12/24 h; however, some authors [81] have shown that repeated urinary samples from the same person tend to have metabolites distributed in the same quartile of concentrations, probably due to an overall little variable basic exposure.

Overall, this study suggests a hypothesis of causality between exposure to certain phthalates and reproductive difficulties, particularly for women with RPL and idiopathic infertility. This finding suggests the need to support prevention initiatives, both in living and working environments, to reduce this type of exposure.

Even if, in some cases, the association between exposure and adverse health effects might not be strong, it seems to be noteworthy for women to reduce contacts with plasticizers in a preventive point of view.

Finally, this is a cross-sectional study which can only produce a hypothesis of causality. Additional case–control surveys and prospective studies are desirable to confirm these emerging hypotheses.

## Figures and Tables

**Table 1:** Occupational studies on phthalate exposure.

Occupational Settings (Number of Subjects)	Biomarkers (Urinary Concentration)	Unit of Measure (Type of Value)	Note	Reference
Hairdressings (*n* = 74)	MEP ^1^ (330) MnBP ^2^(130) MEHP ^3^ (7.5) MEHHP ^4^ (38.0) MEOHP ^5^ (20); sum DEHP ^6^ (65)	µg/L (no creatinine adjusted); mean value	Significantly higher phthalate values compared to controls	[41]
Plastic greenhouse production workers (*n* = 35)	MEP (36.9) MiBP ^7^ (50.8) MnBP (194) MEOHP (17.2) MEHHP (29.7) MEHP (9.76) MBzP ^8^ (0.12)	µg/g creatinine; mean value		[39]
Salesclerks (*n* = 36; 23 in cosmetics area; 4 in perfumes area; 9 in clothing area)	Cosmetics: MEP (91.4) MEHP (53.3) MnBP (220.9) MBzP (19.7) MMP ^9^ (34.4); perfumes: MEP (93.2) MEHP (47.0) MnBP (197) MBzP (17.3) MMP (26.6); clothing: MEP (29.9) MEHP (35.5) MnBP (154) MBzP (12.5) MMP (33.4)	µg/g creatinine; median value in post-shift samples	Significantly higher levels in post-shift than pre-shift for: MEHP and MMP in cosmetics salesclerks; MMP in perfumes salesclerks; MEP in clothing salesclerks	[7]
Waste plastic recycling site workers (*n* = 165)	MMP (24.8) MEP (52.30) MnBP (94.84) MBzP (1.88) MEHP (16.58) MEHHP (76.09) MEOHP (12.53) MnOP ^10^ (0.29	µg/g creatinine; mean value	Higher urinary levels in the exposed group vs. controls for all phthalates except for MnBP	[42]
Hairdressings (*n* = 68)	MEP (201.11) MnBP (103.27) MiBP (61.37) MEHP (10.23) MEHHP (53.37) MEOHP (19.1) sum DEHP (82.70)	µg/L (no creatinine adjusted); mean value	Significantly higher values of MiBP compared to controls	[43]
Plastic manufacturing workers (*n* = 35)	MEHP (13.42) MEHHP (37.53) MEOHP (22.20) sum DEHP (73.15) MiBP (87.17) MnBP (187.21)	µg/L (no creatinine adjusted); mean value	Significantly higher levels compared to controls	[44]
PVC ^12^ production workers (*n* = 82)	MEHP (23.86) MEOHP (66.87) MEHHP (84.56)	µg/g creatinine; geometric mean in post shift samples	Significantly higher concentrations in post-shift than pre-shift	[45]
Plastic manufacturing workers (*n* = 37)	MEHP (35.48) MnBP (108.62) MiNP (13.65) MEP (93.79)	µg/L (no creatinine adjusted); median value	Significantly higher levels compared to controls	[46]
Waste management workers (*n* = 30)	MEHP (15.37) MnBP (71.42) MEP (68.32) MiNP ^11^ (1.47)	µg/L (no creatinine adjusted); mean value	Higher value of MEHP in exposed workers compared to general population	[47]
Workers at flavouring factories (*n* = 71)	MEHP (9.63) MEHHP (14.98) MEOHP (10.18)	µg/g creatinine; mean value	No differences between exposed group and controls	[48]
Plastic manufacturing workers (*n* = 37)	MEHP (35.48) MnBP (108.62) MEP (93.79) MiNP(13.65)	µg/L (no creatinine adjusted); median value	Significantly higher MEHP levels for exposed workers compared to controls	[49]
PVC factories workers (*n* = 89)	MEHP (25.1) MEHHP (97.1) MEOHP (77.4)	µg/g creatinine; geometric mean in post shift for “high exposure”	Post shift levels were significantly higher than pre-shift	[50]
PVC pellet factories workers (*n* = 47)	MEHP (23.5) MEHHP (91.4) MEOHP (75.1)	µg/g creatinine; mean value in post shift for “high exposure”	Significantly higher levels in DEHP exposed workers than controls	[51]

^1^ MEP—mono ethyl phthalate; ^2^ MnBP—mono-n-butyl phthalate; ^3^ MEHP—mono(2-ethylhexyl) phthalate; ^4^ MEHHP—mono(2-ethyl-5-hydroxyhexyl) phthalate; ^5^ MEOHP—mono(2-ethyl-5-hydroxyhexyl) phthalate; ^6^ DEHP—di-(2-ethylhexyl) phthalate; ^7^ MiBP—mono-iso-butyl phthalate; ^8^ MBzP—monobenzyl phthalate; ^9^ MMP—monomethyl phthalate; ^10^ MnOP—mono-n-octyl phthalate; ^11^ MiNP—mono-iso-nonyl phthalate; ^12^ PVC—polyvinylchloride.

**Table 2:** Exclusion and confounding factors for subjects recruited as cases.

Parameter	Confounder	Exclusion Criterion
BMI	X	
Smoke	X	
Alcohol	X	
Genetic diagnosis		X
Urinary creatinine out of normal range WHO		X
Age > 43 years		X
Age between 35 and 43	X	
Chemotherapy treatment	X ^1^	X ^1^
Insulin-dependent diabetes	X	
Hyperthyroidism	X	
Hypothyroidism	X	

^1^ depends on the type and location of chemotherapy.

**Table 3:** Characteristics of the sample.

Characteristics	Women (*n* = 186)
Age (range)	37.6 (29–43)
BMI ^a^ (% of subjects in the class)	
Normal	73.3
Overweight	11.3
Obese	4.3
Underweight	9.1
Unknown	1.6
Present smokers (%)	16.1
Previously smokers (%)	23.1
Alcohol consumption	
Daily	5.9
Weekly	44.6
Monthly	23.7
Never	21.0
Missing	4.8
Residence area (%)	
Urban	79.0
Rural	11.8
Cost	2.2
Industrial	1.1
Urban e industrial	1.1
other	3.7
missing	1.1
Use of plastic containers for fat food storage (%)	
never	17.2
daily	23.1
weekly	38.2
monthly	18.8
missing	2.7
Eating canned food at least weekly (%)	43.5
Eating soya products at least weekly (%)	17.8
Use of scents at least weekly (%)	80.1
Use of nail polishes at least weekly (%)	40.9
Use of hair sprays at least weekly (%)	16.6
Working activity (%)	
Armed forces	0.5
Industrial workers	1.1
Education/leaning area	13.4
employees, professionals, computer operators	47.3
health workers	8.6
Cleaning activity/catering	7.5
Trade	8.1
unemployed	2.2
others	11.3

^a^ BMI: body mass index.

**Table 4:** Infertility factors identified in the female study cohort.

Infertility Factor	Subjects (%)
Ovulatory and endocrine dysfunctions	16.1
Endometriosis	10.8
Idiopathic	28.0
Recurrent Pregnancy Loss (RPL)	9.7
Reduced ovarian reserve	26.9
Reduced ovarian reserve + endometriosis	1.6
Tubal factor	7.0

**Table 5:** Results of urinary phthalate and BPA assays.

µg/g Creatinine		MnBP	MEP	MBzP	MnOP	∑DEHP	BPA
Average		25.70	191.82	5.53	2.14	16.52	0.71
Median		16.29	13.26	2.62	1.25	8.57	0.44
Std. Dev.		38.39	762.55	11.47	2.69	24.30	0.76
Minimum		0.10	0.09	0.06	0.02	0.10	0.01
Maximun		281.71	7574.72	93.39	16.77	187.24	4.71
Percentile	5	1.25	0.77	0.27	0.08	1.15	0.04
	95	108.39	635.08	23.72	8.44	58.26	2.14
% > LOD ^a^		98	72	97	83	98	57

^a^ LOD: limit of detection.

**Table 6:** Data stratification for infertility factors–female sample (*n* = 186).

Infertility Factors	µg/g Creatinine (Average ± SD ^a^)					
	MnBP	MEP	MBzP	MnOP	DEHP	BPA
Ovulatory and endocrine dysfunctions	48.80 ± 71.76	110.05 ± 272.95	9.64 ± 17.12	1.63 ± 1.48	21.91 ± 22.00	0.87 ± 0.53
Endometriosis	25.01 ± 28.82	83.31 ± 96.67	3.69 ± 3.68	1.85 ± 1.54	12.99 ± 14.09	0.51 ± 0.41
Idiopathic	21.15 ± 22.52	230.53 ± 794.17	5.18 ± 9.73	2.95 ± 3.44	16.43 ± 18.17	0.56 ± 0.69
RPL	32.99 ± 42.81	820.45 ± 1929.53	3.53 ± 3.47	1.01 ± 1.13	20.92 ± 42.30	0.83 ± 1.15
Reduced ovarian reserve	15.08 ± 13.58	56.03 ± 139.94	3.36 ± 4.51	1.86 ± 2.33	10.45 ± 13.57	0.69 ± 0.81
Reduced ovarian reserve + endometriosis	14.74 ± 1.39	55.83 ± 90.79	0.62 ± 4.51	1.41 ± 0.62	7.27 ± 5.68	–
Tubal factors	24.82 ± 38.03	75.92 ± 108.48	12.49 ± 25.75	3.37 ± 4.47	29.17 ± 49.55	0.91 ± 0.85
*p* value ANOVA test	0.085	0.104	0.054	0.175	0.032 *	0.142

^a^ SD—standard deviation * statistically significant (*p* < 0.05).

**Table 7:** Stratification of analytical data for different work activities.

	µg/g Creatinine (Average ± SD ^a^)					
Working Activity	MnBP	MEP	MBzP	MnOP	DEHP	BPA
Armed forces ^b^	34.41	3.09	2.74	1.09	9.15	0.05
Industrial workers	18.53 ± 8.66	1.50 ± 0.00	0.61 ± 0.01	2.05 ± 2.57	4.82 ± 6.09	–
Education/leaning area	28.78 ± 57.56	75.50 ± 146.70	4.39 ± 9.97	1.93 ± 3.24	10.50 ± 15.23	0.42 ± 0.60
employees, professionals, computer	24.94 ± 39.14	161.53 ± 813.87	5.51 ± 8.59	2.16 ± 2.60	17.18 ± 25.15	0.63 ± 0.53
health workers	26.87 ± 32.42	484.00 ± 1391.14	3.19 ± 2.84	2.22 ± 2.21	29.73 ± 45.22	0.60 ± 0.59
Cleaning activity/catering	22.42 ± 21.46	218.98 ± 592.62	2.78 ± 2.73	0.94 ± 1.39	12.11 ± 16.16	0.59 ± 0.59
Trade	20.71 ± 14.55	159.65 ± 188.10	8.31 ± 20.92	3.11 ± 2.06	14.73 ± 8.16	1.10 ± 0.48
other	19.84 ± 15.23	98.42 ± 141.51	27.40 ± 44.06	5.09 ± 7.82	17.61 ± 18.93	0.38 ± 0.34
*p* value ANOVA	0.879	0.696	0.228	0.079	0.145	0.015 *

^a^ SD—standard deviation ^b^ 1 subject * statistically significant (*p* < 0.05).

## Data Availability

The data presented in this study are available on request from the corresponding author. The data are not publicly available due to privacy and ethical reasons.
